# NaNuTrap: a technique for *in vivo* cell nucleus labelling using nanobodies

**DOI:** 10.1242/dev.199822

**Published:** 2021-09-01

**Authors:** Zsuzsa Ákos, Leslie Dunipace, Angelike Stathopoulos

**Affiliations:** California Institute of Technology, 1200 East California Blvd, Pasadena, CA 91125, USA

**Keywords:** Nanobody, Nucleus, GFP maturation, Live *in vivo* imaging, Cell tracking, *Drosophila melanogaster*

## Abstract

*In vivo* cell labelling is challenging in fast developmental processes because many cell types differentiate more quickly than the maturation time of fluorescent proteins, making visualization of these tissues impossible with standard techniques. Here, we present a nanobody-based method, Nanobody Nuclear Trap (NaNuTrap), which works with the existing Gal4/UAS system in *Drosophila* and allows for early *in vivo* cell nuclei labelling independently of the maturation time of the fluorescent protein. This restores the utility of fluorescent proteins that have longer maturation times, such as those used in two-photon imaging, for live imaging of fast or very early developmental processes. We also present a more general application of this system, whereby NaNuTrap can convert cytoplasmic GFP expressed in any existing transgenic fly line into a nuclear label. This nuclear re-localization of the fluorescent signal can improve the utility of the GFP label, e.g. in cell counting, as well as resulting in a general increase in intensity of the live fluorescent signal. We demonstrate these capabilities of NaNuTrap by effectively tracking subsets of cells during the fast movements associated with gastrulation.

## Teaser

**Summary:** A nanobody-based method, NaNuTrap, allows early *in vivo* cell nuclei labelling independently of the maturation time of the fluorescent protein and for transforming of cytoplasmic GFP-expressing lines into nuclear-labelled lines.

## INTRODUCTION

The first fluorescent proteins (FPs) were derived from jellyfish in the 1960s (Shimomura et al., 1962) and quickly revolutionized biology (Kremers et al., 2011). Since their discovery, many other variants have been extracted and engineered to improve brightness, widen the colour palette and reduce maturation time (Balleza et al., 2018). The last is especially important during live imaging of transient cell types, and often sets a limitation regarding how quickly a signal can be observed. Options are further restricted with deep-tissue imaging, which requires use of two-photon microscopy commonly supported by a standard Ti:sapphire laser that can be used to excite FPs efficiently in the 700-1000 nm range (Drobizhev et al., 2011; Moulton, 1986). This wavelength range is able to excite a more-limited range of fluorophores, with most having longer maturation times (Drobizhev et al., 2011; Piatkevich and Verkhusha, 2011). At 32°C, 90% of EGFP maturation occurs in about 1 h, with mRFP1 taking a bit longer at ∼80 min and mCerulean a bit faster at ∼50 min (Balleza et al., 2018). At 37°C, 50% maturation is reported by ∼2.5 h for mOrange and ∼1 h for dTomato (Shaner et al., 2004), whereas dsRed versions maturation half-times are in a wide time window ranging from 0.7 to 11 h (Bevis and Glick, 2002). Maturation times vary depending on many factors but generally take longer at lower temperature, such as the 25°C rearing temperature of *Drosophila melanogaster*.

Current genetic methods for cell labelling, as well as emerging image acquisition and analysis methods, now make it possible to track the movement of individual cells in living organisms (McMahon et al., 2008; Progatzky et al., 2013; Stegmaier et al., 2016) and to automatically track differentially FP-labelled cells to reconstruct cellular level interactions (Prasad and Montell, 2007; Smutny et al., 2017; Theveneau et al., 2013). However, many developmental processes are short-lived, which makes labelling cells a challenging task. For example, mesoderm spreading in *Drosophila* takes only about 1 h (see Fig. 2A; Clark et al., 2011; Leptin, 2005; Sun et al., 2020) and even with a strong mesoderm-specific zygotic driver, such as the *twist* (*twi*) enhancer, GFP expression is not detectable early enough to illuminate the process from its beginning (Clark et al., 2011).

To capture mRNA synthesis – the earliest readout of the activity of a gene – live, the MS2-MCP system (Bertrand et al., 1998; Forrest and Gavis, 2003) was tailored for use in *Drosophila* (Garcia et al., 2013; Lucas et al., 2013). This system overcomes the challenge of waiting for FP maturation by depositing the parts of the system carrying the FP maternally (i.e. MCP-FP fusion proteins). However, this system is not particularly suitable for cell tracking as MCP-FP signal associated with nascent transcription presents as small spots within the nucleus, which does not support robust identification or tracking of individual nuclei over time, especially if cells are moving. We therefore focused our approach on alternative methods that permit labelling of entire nuclei.

Nanobodies (Hamers-Casterman et al., 1993) are small single-domain antibodies usually derived from camelids or sharks (Könning et al., 2017); designed ankyrin repeat proteins (DARPins) are small synthetic proteins that also can engage in selected binding. When these molecules, nanobodies or DARPins, are fused to FPs, they have been used successfully to label specific cell compartments, both in cell culture (Rothbauer et al., 2006) and in living organisms (Harmansa and Affolter, 2018). Nanobodies have also been applied in assays to sequester, and therefore interfere with, secreted protein distribution, indicating that they are indeed robust and specific binders (Harmansa et al., 2015). In addition, these molecules have also been useful in tracking subcellular translocation events; e.g. in mammalian cell culture, GFP nanobody was fused to a nuclear localization sequence (NLS) (Kalderon et al., 1984) and used to detect translocation of another GFP-fusion protein into the nucleus (Kirchhofer et al., 2010). In a more recent study in *Drosophila*, CRISPR gene editing was used to generate FP-specific nanobody fused to transcription factors (TFs), thereby permitting observation of transcription factor dynamics live (Bothma et al., 2018). This particular method also involved use of maternally deposited FPs in embryos to overcome the limitation imposed by maturation time of FPs in observing early zygotic gene expression. As these TFs localize within the nucleus, the FPs were also enriched in the nucleus, allowing labelling of nuclei in early embryos but with the downside that making such TF-nanobody fusion transgenic can be labour intensive and challenging, with the added possibility that fusion proteins alter TF function.

Here, we introduce a new nanobody-based labelling method to *Drosophila*, in which a GFP-specific nanobody tagged with three nuclear localization sequences (3×NLSs) draws GFP into the nucleus, permitting efficient cell labelling *in vivo*. This Nanobody Nuclear Trap (NaNuTrap, NNT) is expressed using the existing *Drosophila* Gal4/UAS (Brand and Perrimon, 1993) system and can therefore be used to label specific cell groups using existing Gal4 lines without additional genetic modification. When coupled with fly stocks that maternally deposit GFP, this new tool accelerates nuclear labelling in early embryos by eliminating the lag usually associated with FP maturation. NaNuTrap also has the potential to convert cytoplasmic GFP expressed in any extant transgenic fly line into a nuclear label, again without additional genetic modification.

## RESULTS AND DISCUSSION

### NaNuTrap allows early labelling of nuclei *in vivo*

**Fig. 1.**
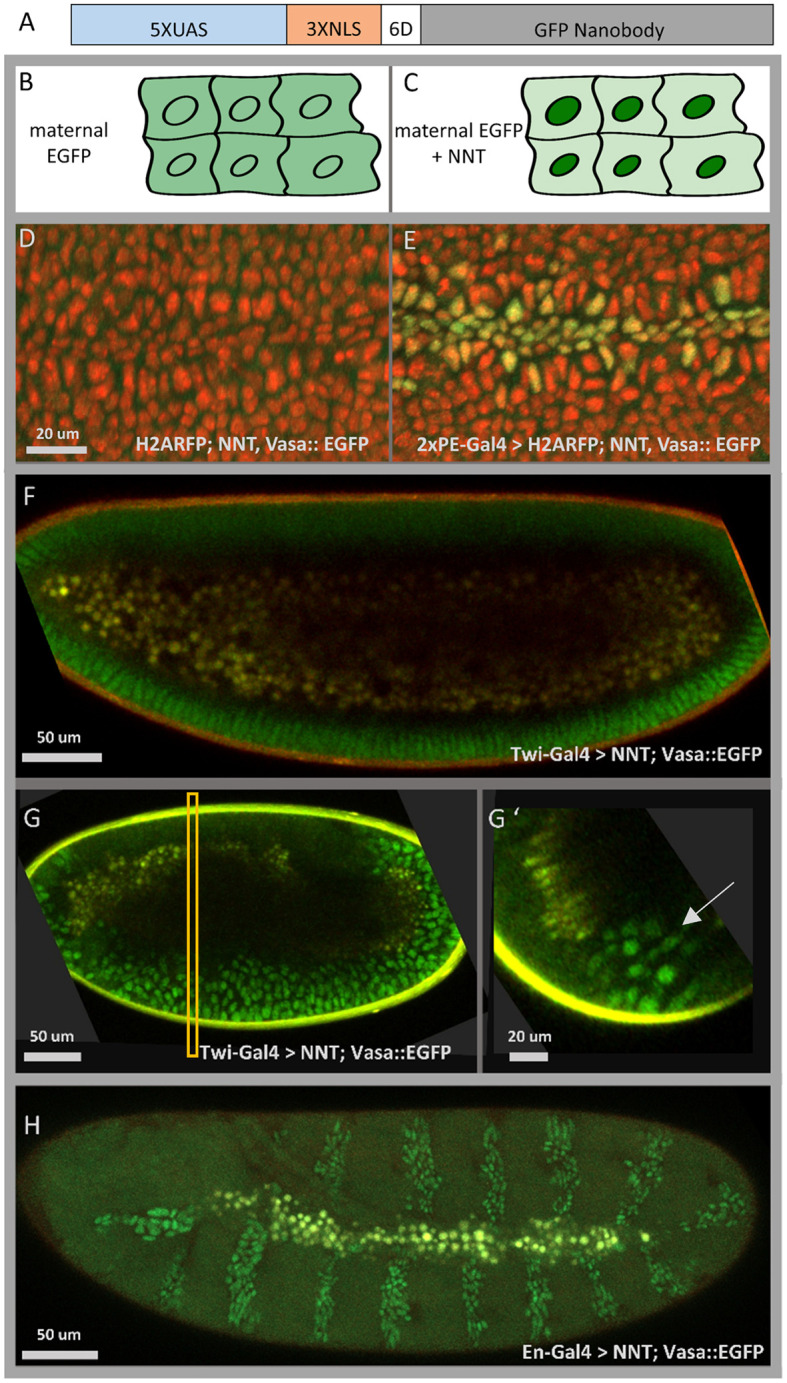
**The NaNuTrap (NNT) method.** (A) The 5×UAS::NNT construct: a GFP nanobody is fused to three nuclear localization sequences (3×NLS) with a six aspartate (6D) linker sequence and expression driven by the Gal4/UAS system (5×UAS). (B,C) Schematic illustrating the NNT method. (B) Maternally deposited GFP is distributed evenly in the embryo. (C) When the nanobody construct NNT is also expressed, it drives the already matured GFP into the cell nuclei. (D,E) Microscope stills from live *in vivo* imaging sessions in which the maternal driver Vasa is used to deposit GFP (Vasa::EGFP, green) in a His2Av-mRFP1 labelled (red) embryo (stage 10) (D); however, nuclear signal from GFP (green) is observed only when co-expressed with the NNT construct (E). (F) In the presence of maternally deposited GFP, the NNT construct, when expressed zygotically in the mesoderm and midline using the Twi-Gal4 driver, can localize GFP into the nuclei of the presumptive mesoderm cells in the cellular blastoderm (F, stage 5). (G,G′) Two-photon images of the collapsing furrow (stage 7/8); embryo genotype is the same as in F. Longitudinal section (G) and cross-section (G′) of the same 3D reconstructed image. Yolk autofluorescence appears in yellow, while signal from GFP-expressing nuclei is green. The rectangle in G indicates the position of the cross-section view shown in G′. Arrow indicates mesoderm cell nuclei. (H) Microscope still from a live *in vivo* imaging session (see Movie 4) of an embryo expressing NNT using the En-Gal4 driver; EGFP was deposited maternally.

We have created a UAS reporter line (5×UAS) that expresses a GFP nanobody (Bothma et al., 2018; Kirchhofer et al., 2010) fused to a 3×NLS sequence (Chertkova et al., 2020) able to localize maternally deposited GFP into the nucleus. Variables tested include two different linker sequences [i.e. six aspartates (6D) and six glycines (6G)] shown to differentially affect nuclear localization (Martin et al., 2015), as well as the relative position of the NLS and nanobody sequences. We found that the 6D and 6G linker sequences work equivalently, whereas placement of the NLS at the N terminus of the nanobody sequence drives nuclear localization more efficiently than when it was placed at the C terminus (data not shown). The construct, N-terminal 3×NLS followed by a 6D linker and fused to GFP nanobody, is henceforth referred to as NaNuTrap (NNT) (Fig. 1A).

A fly line was generated that expresses GFP maternally through Vasa::EGFP (Bothma et al., 2018) and carries the NNT construct (5×UAS::NNT; Vasa::EGFP); this line was combined with various Gal4 drivers to support targeted expression of NNT using standard crosses. In embryos derived from this cross, we found that our NNT construct efficiently transfers maternal GFP into the nucleus in a GAL4-dependent manner (Fig. 1E,F), thereby making nuclei bright enough to detect the tissue-specific expression of different drivers live through confocal (Fig. 1E,F,H; e.g. see Movie 1) and two-photon microscopy (Fig. 1G,G′).

**Fig. 2.**
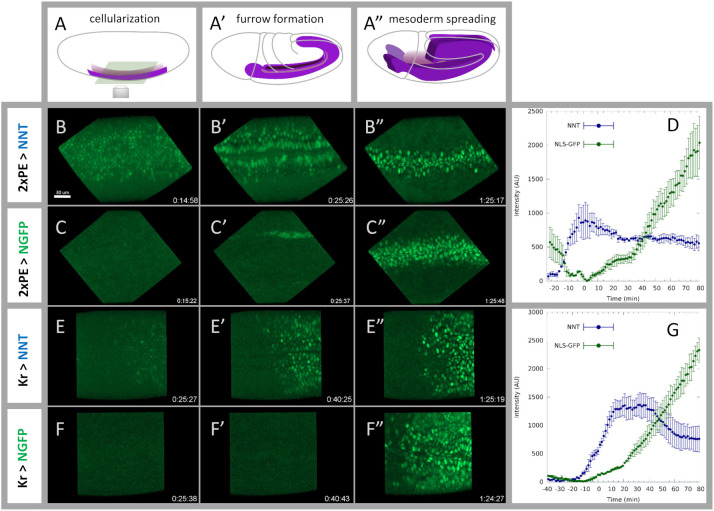
**NaNuTrap-driven fluorescent signal precedes NLS-GFP signal.** (A-A″) Schematic of the developmental stages corresponding to snapshots from movies shown in the same column: stage 5, cellularization (A); stage 7, furrow closure (A′); and stage 9, mesoderm spreading (A″). (B-C″) Snapshots from live movies: 2×PE-Gal4 driver is used to label mesoderm by driving the expression of the NNT construct (B-B″) or a NLS-GFP construct (C-C″). NNT-driven signal appears during invagination, whereas NLS-GFP signal is still not detectable at furrow closure. Movies start 25 min before ventral furrow closure. (D) Mean intensity of GFP signal shown over time. NNT-driven signal intensity is shown in blue; NLS-GFP is shown in green (*n*=3 embryos, error bars indicate s.e.m). (E-F″) Snapshots from live movies: Kr-Gal4 driver is used to label ectodermal cells by driving the expression of the NNT construct (E-E″) or a NLS-GFP construct (F-F″). Movies start 40 min before furrow closure. (G) Mean intensity of GFP signal shown over time. NNT-driven signal intensity is shown in blue; NLS-GFP is shown in green (*n*=3 embryos, error bars indicate s.e.m). All curves (D,G) were shifted in time such that t=0 corresponds to furrow closure. Time is shown in hh:mm:ss format in movie snapshots.

To evaluate the timing and intensity of the signal from this new NNT system versus traditional labelling approaches, we drove either NNT (with maternally deposited GFP) or a standard NLS-GFP transgene with the same GAL4 and compared the nuclear fluorescent signal (Fig. 2, Fig. S1). Embryos were imaged live (Movies 1 and 2) and the change in fluorescent intensity over time was measured (see Materials and Methods). Two different Gal4 drivers that are expressed during gastrulation (Fig. 2A-A″) were used to express either NNT or NLS-GFP: a mesoderm-specific Gal4, 2xPE-Gal4 (Fig. 2B-D); and an ectoderm-specific Gal4, Kr-Gal4 (Fig. 2E-G). We were able to observe fluorescent signals 31 min and 15 min earlier using the NNT system with 2×PE- and Kr-Gal4, respectively, when compared with using the NLS-GFP (see Materials and Methods). The intensity of the signal driven by the NNT system also increased more quickly. NLS-GFP signal was only able to match the intensity level of the NNT about 60 min after initial detection of the NNT signal (60 and 59 min for the 2×PE and Kr drivers, respectively; see supplementary Materials and Methods). Many developmental processes are very fast, e.g. mesoderm spreading, which takes only about 1 h to complete (Sun et al., 2020). As live imaging is ideal for such quick, dynamic processes, the ability to rapidly increase fluorescent signal while labelling specific cell groups is crucial.

This method could also be used to generate marked balancer lines, usable at earlier stages, by introducing the NNT and Vasa::EGFP constructs into existing, widely used fluorescent balancer chromosome lines used in *Drosophila*, thereby permitting discernment of homozygous versus heterozygous genotypes. Balancers using Kr-Gal4, such as TKG (TM3, Kr-Gal4 UAS-GFP), are detectable at stage 9 (Casso et al., 2000); those using Twi-Gal4 and 2×EGFP (Halfon et al., 2002) are detectable earlier, at stage 8 (Movie 3). As shown in Movie 3, upon co-expression of NNT and maternally loaded GFP, presence of these balancers can be detected earlier, during cellularization at stage 5. This is highly advantageous for distinguishing zygotic genotypes using live-imaging approaches.

Our analysis also revealed how the maternal GFP signal, the limiting factor for NNT functionality, plateaus over time (Fig. 2D, G). We tested a number of Gal4 lines (Fig. S2, Movie 4) and found that the NNT system also worked with drivers expressed after gastrulation, e.g. En-Gal4 (Fig. S2, Movie 4). Using the NNT system to visualize En-Gal4 action, we found that expression was initiated starting at stage 9 following germ band extension. This indicates that, although the NNT uses maternally deposited GFP, the label perdures into embryonic development and can therefore be used to speed up and intensify visualization of expression supported at later stages. Nevertheless, because zygotically expressed NLS-GFP can potentially accumulate over time, depending on the specific driver, it would be ideal to have both maternally deposited and zygotically expressed fluorophores available to study longer developmental processes. For such purposes, we generated a fly stock containing both the NNT system and the NLS-GFP transgene, which combines the advantage of early nuclear labelling with ongoing accumulation of nuclear GFP signal over time (Movie 5).

### Other applications: making cytoplasmic GFP nuclear with NaNuTrap

**Fig. 3.**
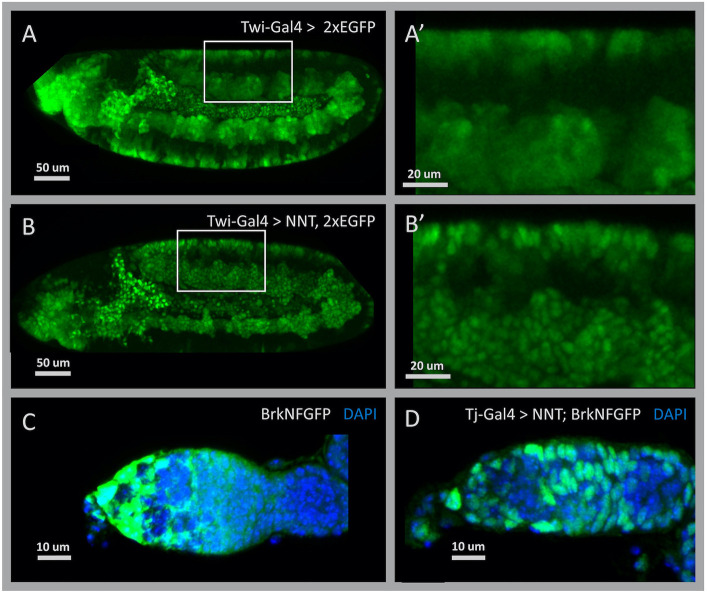
**NaNuTrap efficiently transfers zygotically expressed cytoplasmic FP into the nucleus *in vivo*.** (A) TTG (Twi-Gal4, UAS-2×EGFP) balancer line embryo at stage 11. A large amount of EGFP is expressed in mesoderm derivative cells. (A′) Higher magnification of the area indicated in A. (B) Additional to cytoplasmic EGFP, NNT is also expressed by the Twi-Gal4 driver of the TTG (TM3, Twi-Gal4, UAS-2×EGFP) construct in the embryo shown at stage 11. (B′) Higher magnification of the area indicated in B. (C) Fixed tissue sample of germarium, anterior to the left, showing GFP expression driven by the large reporter construct BrkNFGFP (green; Dunipace et al., 2013) and nuclei stained with DAPI (blue). (D) The BrkNFGFP reporter combined with NNT and a pan follicle cell driver, Tj-Gal4, show distinct nuclear localization of GFP within cells.

In addition to early nuclear labelling, we also investigated whether the NNT construct could be used to transfer not only a limited amount of maternal GFP but also the potentially larger amounts of GFP expressed by some zygotic drivers into the nucleus. This would allow one to convert the cytoplasmic GFP expressed in any of the thousands of available transgenic fly lines into a nuclear marker by simply crossing them to NNT, thereby facilitating counting or tracking of cells without any genetic modification necessary. In order to test this, we crossed the NNT line to the TTG balancer stock, which expresses cytoplasmic GFP in the mesoderm, producing a very bright signal (TM3, twi>Gal4 UAS-2×EGFP) (Fig. 3A,A′; Halfon et al., 2002). Indeed, we find that the NNT construct can efficiently transfer sufficient amounts of GFP into the nucleus to permit nuclear detection in the mesoderm (Fig. 3B,B′). This nuclear enrichment functionality could also be used to concentrate otherwise weak or diffuse cytoplasmic GFP signal and permit more-robust identification of expressing cells or to distinguish them from auto-fluorescence.

**Fig. 4.**
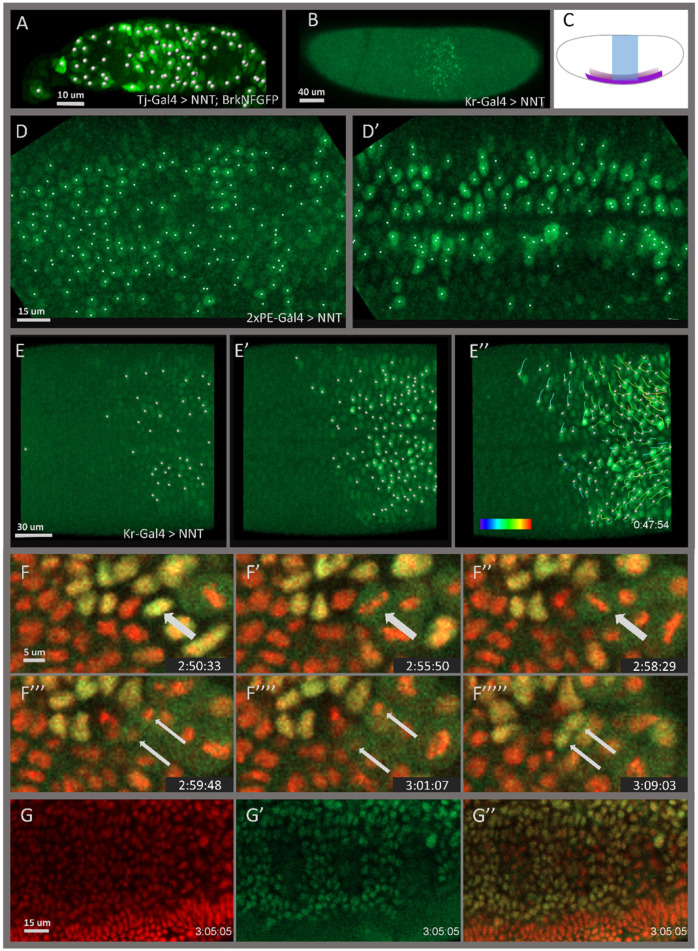
**Cell nuclei segmentation and tracking in embryos labelled using the NaNuTrap method.** (A) Cell nuclei segmented automatically, in an immunostained tissue sample of the germarium from a BrkNFGFP reporter line that was turned into a nuclei-labelled line with NNT. (B,C) Kr expression pattern in early stage 7 embryo: snapshot from live movie of an embryo labelled with NNT using the Kr-Gal4 driver (B) and a schematic of expression of Krüppel (Kr) (blue) and 2×PE/*twist* (purple) (C). (D,D′) Cell nuclei segmented in live movies of embryos that are expressing NNT using the 2×PE-Gal4 driver to label mesoderm and midline (Movie 1) at stage 5 cellularization (D) and at furrow closure (D′). (E-E″) Cell nuclei segmented in live movies of embryos that are expressing NNT using the Kr-Gal4 driver to label ectodermal cells (Movie 2) at stage 5 cellularization (E) and at furrow closure (E′). Cells are also tracked automatically during the time period when conventional cell labelling methods are not labelling cell nuclei efficiently (Movie 2). Colour bar indicates the average speed of the tracks (0-4.5 µm/min) (E″). Cells were segmented and tracked automatically using Imaris spot detection and tracking functions. (F-F″′″) Loss of NNT signal is an indicator of cell division. Snapshot from a live movie (Movie 7) of a His2Av-mRFP1 embryo (stage 10) labelled with NNT using the Twi-Gal4 driver, showing a cell before division (F), in different cell division phases – metaphase (F′,F″), anaphase (F″′) and telophase (F″″) – and after division (F″′″). Arrows indicate the same cell (wide arrow) and its daughter cells (two thin arrows). (G-G″) Cell division pattern in the mesoderm of a *Drosophila* embryo (stage 10) during the 3rd mesodermal division. Genotype is the same as described in F. When NNT labelling is shown alone (G′) or superimposed onto His2Av-mRFP1 signal (G″), tissue level division patterns can be visualized in the mesoderm. EGFP was deposited maternally in embryos shown in B,D-G″.

The availability of *Drosophila* lines expressing fluorescently tagged proteins generated through enhancer trapping (Kelso et al., 2004; Nagarkar-Jaiswal et al., 2015; Singari et al., 2014), user-defined FP reporter constructs (e.g. Kudron et al., 2018) and homologous recombination or CRISPR-mediated introduction of FP-tags into genes (Dunst et al., 2015; Li-Kroeger et al., 2018) is growing exponentially. Many of these fluorescent reporters are cytoplasmically expressed and the signal is too low to be detectable live, or too diffuse to be reliably localized in fixed tissues. To demonstrate how the NNT system can enhance the utility of GFP reporters in such scenarios, we turned to a large GFP reporter construct we constructed in a previous study designed to capture expression of the gene *brinker* (*brk*) in the early embryo (Dunipace et al., 2013). We recently found that this construct also supports expression of *brk* in the ovary, which is a dense collection of interconnected cells, and native reporter GFP expression was present throughout the germarium in a diffuse pattern (Fig. 3C). By driving NNT with Tj-Gal4, which is expressed in the somatic follicle cells that encapsulate the developing germline, cytoplasmic GFP is efficiently concentrated in nuclei, permitting identification and counting of follicle cells that was not previously possible with this reporter (Figs 3D and 4A). NNT would similarly be expected to facilitate visualization of the large number of endogenously GFP-labelled *Drosophila* proteins (Sarov et al., 2016; Kina et al., 2019) by altering the location of the endogenous proteins to support early nuclear localization; however, doing so could affect protein function and cause mutant phenotypes.

### Concluding remarks

We developed NaNuTrap, specifically, for automated cell segmentation and tracking to investigate cellular movements in the fast-developing early *Drosophila* embryo. To determine whether NaNuTrap-generated signal permits cell tracking analyses, we captured movies of live embryos in which NNT expression was driven by 2×PE and Kr-Gal4 (Fig. 4B,C; Movies 1 and 2) and tested whether automatic segmentation could be applied to NNT-localized, maternally deposited GFP. Although signal initiation was variable at the cellularization stage (Fig. 4D,E), segmentation became reliable shortly thereafter – by the time the mesoderm invaginates (i.e. furrow formation) (Fig. 4D′,E′) – and remained suitable for automatic tracking (Fig. 4E″, Movie 6) throughout the fast phase of germ band elongation. During this time period, signal has proved to be insufficient in the past to support segmentation and tracking using conventional labelling techniques for these cell types (Fig. 2C′,F′, Movies 1 and 2). In addition, cells lose signal during cell division but quickly regain it (Fig. 4F-F″′″, Movie 7). Although an additional implication of this result is that cell lineages cannot be followed solely using the NNT-based labelling approach, we found that NNT also helps visualize cell division irregularities in the labelled tissue (Fig. 4G-G″). While cell division phases of individual cells can be determined based on histone labelling (Fig. 4G), tissue level patterns became more apparent when the NNT signal was superimposed (Fig. 4G″). For lineage tracing, a similar method could potentially be developed in which, instead of the 3×NLS, a histone protein or a histone nanobody (Jullien et al., 2016) is fused to the GFP nanobody such that it promotes association with the chromatin during cell division and supports continuous tracking.

In summary, we have taken an approach that uses NLS and a nanobody to localize GFP into the nucleus, and have applied it, together with a source of maternally deposited GFP, to use for *in vivo* labelling in *Drosophila*. In order to make it easily adaptable, we created our NNT transgenic lines as UAS reporter lines to facilitate labelling of different cell types. We were able to accelerate labelling compared with traditional labelling methods that rely on zygotic GFP expression, as labelling initiated 15-30 min earlier and for the first hour was brighter. Our method based on the UAS system can be used with the available large number of existing Gal4 lines through a single cross, in a matter of a couple of weeks; a related approach, LlamaTag, is more complicated, as creation of nanobody fusions to transcription factors (or other proteins) takes much longer, is labour intensive and also may affect function of the tagged endogenous protein. Another way to tackle the problem of long FP maturation times is to develop fast-maturing FPs. Recently, a method was described in which fast-maturing FP mNeonGreen was fused to NLSs and other sequences that boost efficiency of translation, and used to assay early zygotic expression in *Drosophila* embryos (Ceolin et al., 2020). This other approach is still dependent on the maturation time of the FP (for mNeonGreen has a maturation half time of ∼7-10 min), is currently not compatible with the Gal4 system and is less suitable for 2P imaging than GFP (Molina et al., 2017); however, it has an advantage over NNT in that it is not dependent on maternal GFP. The NNT approach presented here has the potential to be expanded to other fluorescent proteins with favourable spectral properties but the longer maturation times of which have precluded their use for live imaging in the past. In addition, in combination with existing Gal4-based FP balancer lines, NNT allows earlier visualization of commonly used FP balancer chromosomes to facilitate their use in younger embryos. Last, the created NNT lines are capable of the transformation of GFP expressed in existing transgenic fly lines, from a cytoplasmic into a nuclear marker that allows for cell segmentation that would not be possible with currently available methods. Here, we have focused on how NNT can support cell tracking and earlier visualization of FPs in embryos but similar ‘fast-tracking’ of FP can be supported at later stages in *Drosophila*, as well as in other animals that support the Gal4-UAS expression system (Distel et al., 2009; Ovchinnikov et al., 2008; Wang et al., 2017).

## MATERIALS AND METHODS

### *Drosophila* stocks and genetics

5×UAS::NNT, this study, was combined with Vasa::EGFP (Bothma et al., 2018) to create *5×UAS::NNT; Vasa::EGFP*. Virgins were collected from *5×UAS::NNT; Vasa::EGFP* and crossed to males from 2*×*PE (y[1] w[*]; P{w[+mC]=GAL4-twi.2xPE}2,:BDSC_2517), Kr (*P{w[+mC]=GAL4-Kr.C}10o/TM3*,Sb[1], BDSC_58800), En (w[*]; P{w[+mC]=stg-lacZ.beta-E2.2}2; P{w[+m*]=en-GAL4.U}3, BDSC_83350), Prd (w[*]; P{w[+mW.hs]=GAL4-prd.F}RG1/TM3, Sb[1], BDSC_1947) or Hairy (w[*]; P{w[+mW.hs]=GawB}h[1J3], RRID:BDSC_1734) Gal4 lines and to TTG balancer line (*TM3,Twi-Gal4,2×EGFP*) (Halfon et al., 2002). For flies expressing only NLS-GFP, virgins were collected from UAS::NLS-GFP (*w[1118]; P{w[+mC]=UAS-GFP.nls}8*, BDSC_4776) and crossed to 2*×*PE-Gal4 or Kr-Gal4 males. To express both NNT and NLS-GFP in the mesoderm, fly stocks *2×PE-Gal4; Vasa::EGFP* and *5xUAS::NNT; UAS::NLS-GFP* were created. Embryos were collected from crossing virgins from *2×PE; Vasa::EGFP* with males from the *5×UAS::NNT; UAS::NLS-GFP* line. To visualize cell division, we generated line *His2Av-mRFP1; NNT, Vasa::EGFP* (using line w[*]; P{w[+mC]=His2Av-mRFP1}II.2, BDSC_23551), crossed it to the Twi-Gal4 (*P{w[+mC]=GAL4-twi.G}108.4, w[1],* BDSC_914) driver, and collected the resulting embryos. The BrkNFGFP fly stock has been published previously (Dunipace et al., 2013).

### Embryo handling and live imaging

Embryos were manually dechlorinated and mounted on a heptane glue slide in a drop of water. Confocal images were recorded using a Zeiss Axio Imager Z2 confocal microscope either with a C-Apochromat 40×/1.2 NA or with a LCI Plan-Neofluar 25×/0.8 NA water dipping objective lens. Images were taken in 15-20 *z*-stacks separated by 3 µm. Videos were acquired with a frame rate between 75 and 93 s. GFP was excited at 488 nm with 4-5% of laser power. To visualize yolk and separate it from GFP signal, some movies were also excited at 561 nm with 1% laser power. Gain was set to 550 V, the pinhole to 100 µm and pixel dwell time to 0.76 or 1.52 µs. Images analysed in Fig. 2 were recorded with the same settings. Two-photon images were recorded using a Zeiss LSM 880 microscope and a LCI Plan-Neofluar 25×/0.8 NA water dipping objective lens. GFP was excited at 860 nm with 7% of laser power, two times averaging and 2.06 µs pixel time, and detected using the NDD detector. 102 *z*-stacks were taken separated by 1.1 µm.

### Ovary staining

Ovaries were collected and stained with GFP antibody (Rockland, 600-101-215, 1:5000) and anti-goat 488 (Life Technologies, A11055, 1:400) as described previously (Zimmerman et al., 2013), and mounted in SlowFade Gold with DAPI (Invitrogen, S36939).

### Plasmid construction and injection

NNT design includes three copies of an NLS sequence (PKKKRKV) (Kalderon et al., 1984) linked with aspartic acid residues to create 3×NLS [as previously described by Chertkova et al. (2020)]. The 3×NLS was fused with a 6D or 6G linker to codon optimized GFP nanobody (Bothma et al., 2018). For further details of the design, see the supplementary Materials and Methods.

### Image processing and data analysis

Images were analyzed using ImageJ. *Z*-projections of the 20 *z*-stacks of the green channel were created using the sum slices function. Images were rotated by marking the midline and rotating into a horizontal position with the head pointing to the left. Background was subtracted using the rolling ball function with 50 µm radius. A rectangle was selected and placed close to the midline while avoiding yolk signal (120×60 µm for 2×PE-Gal4, and 40×140 µm for Kr-Gal4) to measure and plot the mean intensity value versus time curve using the Plot *z*-axis profile command. The resulting intensity/time curves were shifted in time, such that the 0 time point corresponded to furrow closure (determined based on the brightfield and green channel images) and 0 intensity corresponded to minimal intensity detected. For details, see the supplementary Materials and Methods.

## Supplementary Material

10.1242/develop.199822_sup1 Supplementary information
