# Supplementary material for: NaNuTrap: a technique for in vivo cell nucleus labelling using nanobodies
Source: Development. 2021 Sep 1;148(18):dev199822. doi: 10.1242/dev.199822 (PMC10656463; doi:10.1242/dev.199822)
Supplement: Supplementary information [file develop-148-199822-s1.pdf]

Supplemental methods

Plasmid construction and injection

We have designed NNT as follows: NLS sequence (PKKKRKV) (Kalderon et al., 1984) was linked together 3 times with aspartic acid to create 3xNLS [as described before by (Chertkova et al., 2020)]. The 3xNLS was fused with a 6D or 6G linker to codon optimized GFP nanobody (Bothma et al., 2018). The 3xNLS-6D-GFP nanobody (NNT) sequence was ordered from IDT as gBlocks and inserted into pUASt-attB (5xUAS) vector, a gift from Konrad Basler (Bischof et al., 2007), using EcoRI and XbaI cutting sites. NNT constructs were injected by Rainbow Transgenic Flies, Inc. using site specific injection into the second (6D version, R8622) and third (6D and 6G versions, attp40w) chromosomes.

Image processing and data analysis

Intensity over time curve was calculated in the following way. Images were analyzed using ImageJ. Z projections of the 20 Z-stacks of the green channel were created using the sum slices function. Images were rotated by marking the midline and rotating into a horizontal position with the head pointing to the left. Background was subtracted using the rolling ball function with 50 µm radius. A rectangle was selected and placed close to the midline while avoiding yolk signal (120x60 µm for 2xPE-Gal4, and 40x140 µm for Kr-Gal4) to measure and plot the mean intensity value versus time curve using the Plot Z-axis profile command. For Kr-Gal4, we placed the rectangle closer to the posterior side where the signal was still detectable at the end of the movie. The resulting intensity/time curves were shifted in time, such that the 0 time point corresponds to furrow closure (determined based on the brightfield and green channel images) and 0 intensity corresponds to minimal intensity detected. We averaged data from three embryos. To determine the difference in timing of signal appearance we defined a 150 A.U. as minimum intensity (a threshold

Development • Supplementary information

when by eye we could see signal) and calculated intersection of the average intensity curves and the 150 A.U. line. Signal was starting at time (average ± SD, n=3 embryos) relative to furrow closure (t=0), for 2xPE-Gal4 driver, 2xPE_NNT = -18 ± 2.2 min, 2xPE_NLS_GFP = 14 ± 5.5 min and for Kr-Gal4 driver, Kr_NNT= -11 ± 2.3 min, Kr_NLS_GFP = 4 ± 3.9 min). Similarly we have calculated the intersection point of the two average curves (NNT and NLS_GFP) to determine the time when they were reaching the same intensity level (average curves were intersect at 42 min and at 48 min after furrow closure (t=0) respectively for 2xPE and Kr-Gal4). Intersections were calculated using Python and data were plotted in Gnuplot.

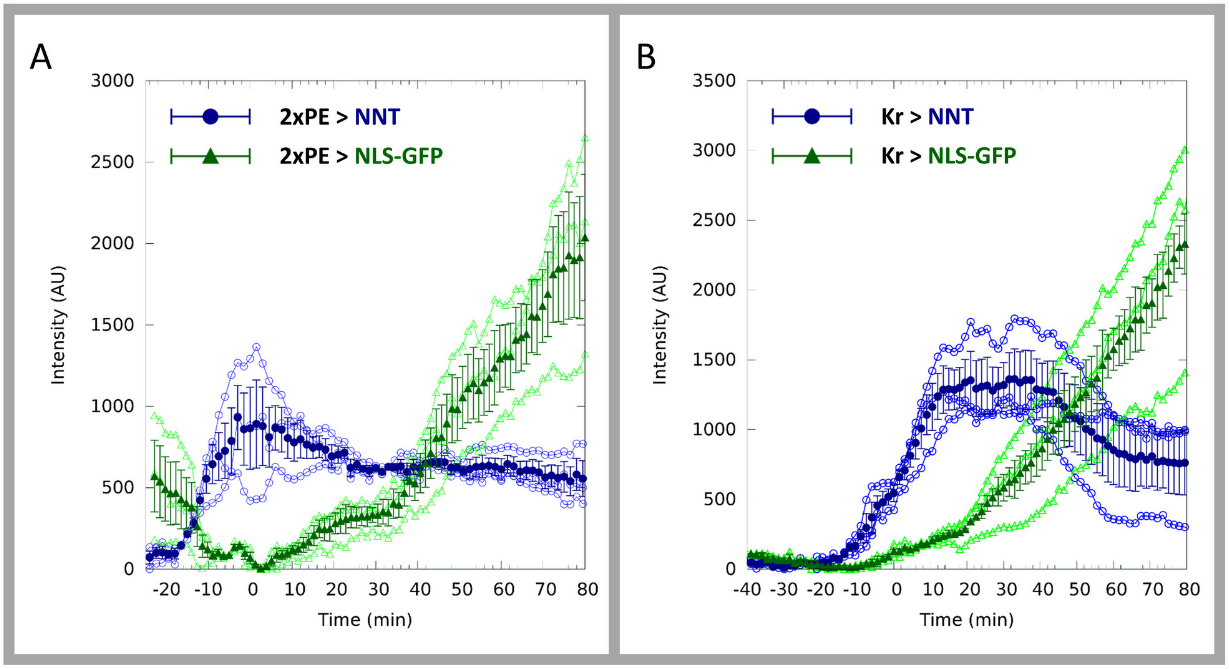

Fig. S1. Individual intensity curves for figure: NaNuTrap driven fluorescent signal precedes NLS-GFP signal (Fig. 2D, G). (**A, B**) 2xPE-Gal4 (A) and Kr (B) drivers were used to label mesoderm and ectodermal cells respectively by driving the expression of the NNT construct (blue) or an NLS-GFP construct (green), see also Fig. 2. Individual intensity curves for embryos (open marks) and mean intensity of GFP signal shown (closed marks) over time. NNT-driven signal intensity shown in blue, NLS-GFP shown in green (n=3 embryos, error bars s.e.m.). All curves were shifted in time such that t=0 time point corresponds to furrow closure.

Development • Supplementary information

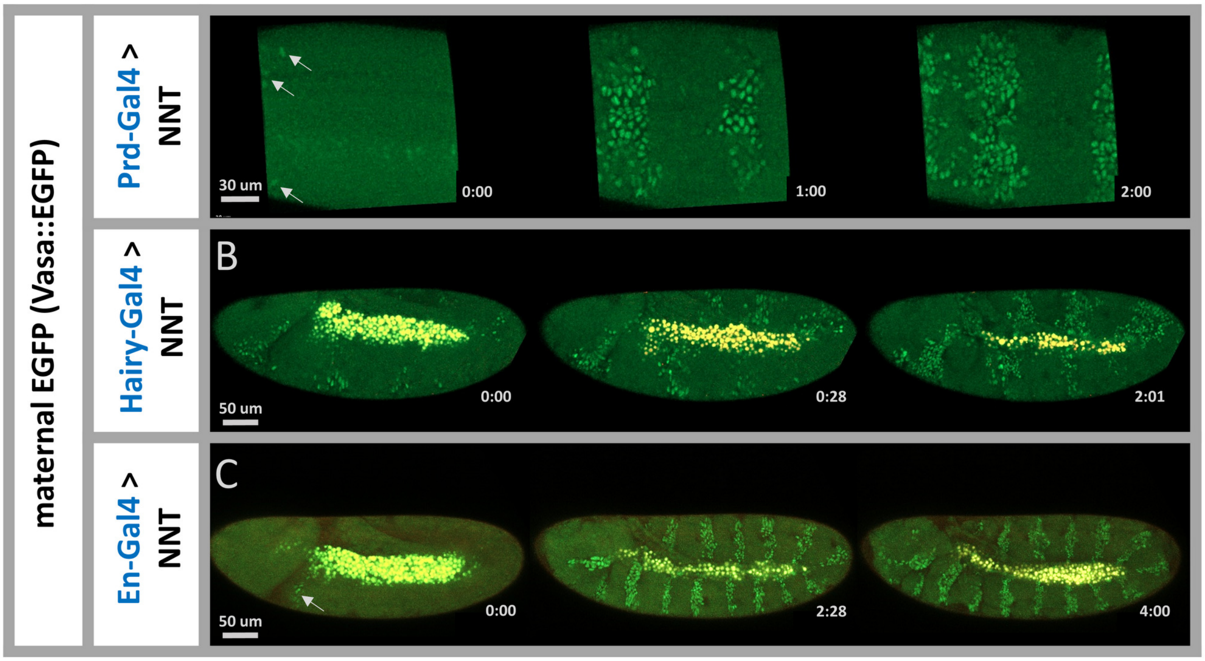

Fig. S2. Labelling cell nuclei using NaNuTrap with Gal4 drivers

(A,B,C) Snapshots from live movies of embryos expressing NNT using the PrD-Gal4 (A), Hairy-Gal4 (B) and En-Gal4 drivers (C). Images show representative time points of the movies starting when the signal was first detected. EGFP was deposited maternally. Embryos were imaged from the ventral (A) or lateral (B,C) side, with anterior to the left. Note that the autofluorescent signal from the yolk is yellow (B,C) and can be separated from the GFP signal shown in green (see Methods). Arrows indicate cell nuclei in frames associated with the initial signal present only in a few cells. Time is shown in hh:mm format.

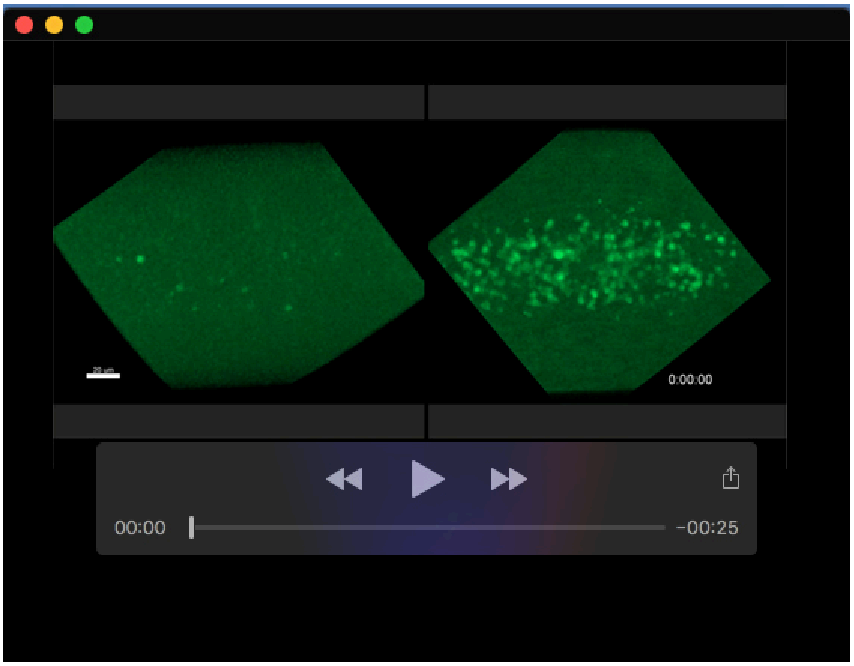

Movie 1. Early cell nuclei labelling using NNT construct and 2xPE-Gal4 driver. 2xPE-Gal4 is driving the expression of the NNT construct with EGFP deposited maternally (left), or the NLS-GFP construct (right) to label the mesoderm. Time-lapse movies start during cellularization at stage 5 (25 min before furrow closure). In this and subsequent movies, the embryo was imaged from the ventral side, with anterior to the left.

Development • Supplementary information

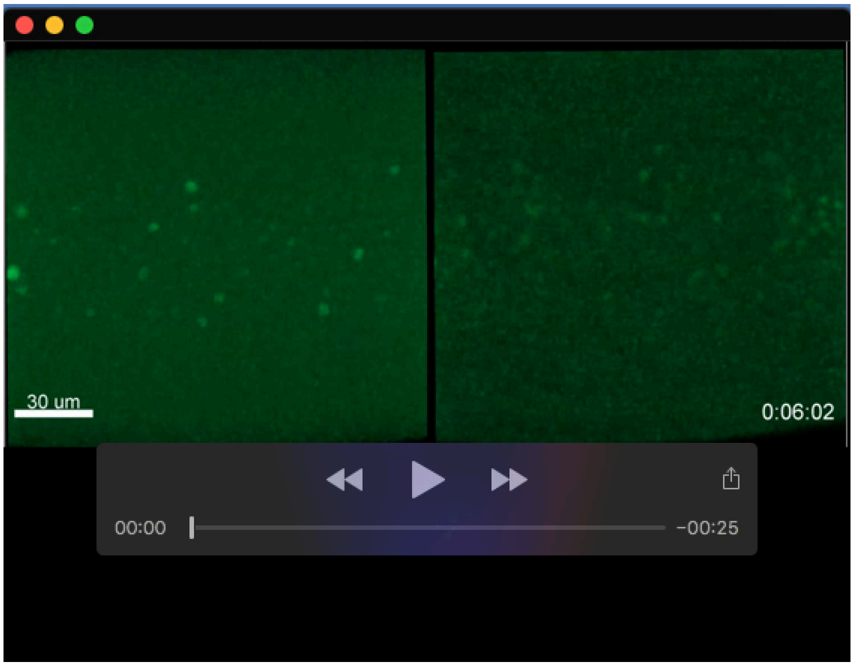

Movie 2. Early cell nuclei labelling using NNT construct and Kr-Gal4 driver. Kr- Gal4 is driving the expression of the NNT construct with EGFP deposited maternally (left), or the NLS-GFP construct (right). Time-lapse movies start during cellularization at stage 5 (i.e. 40 min before furrow closure).

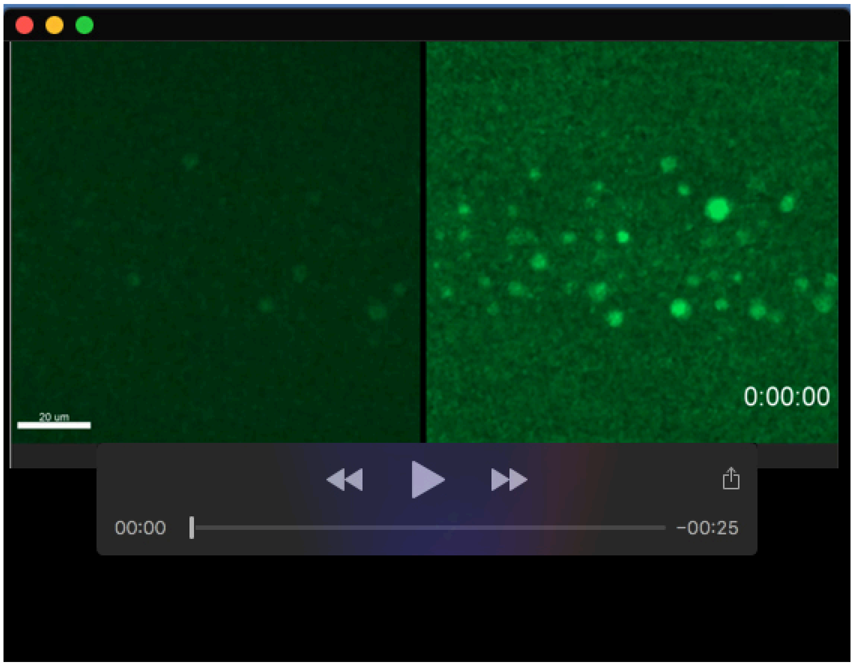

Movie 3. Early expression with the help of NNT using the existing TTG balancer line. Twi-Gal4 of the TTG (Twi-Gal4, 2xEGFP) balancer line is driving the expression of the NNT construct with EGFP deposited maternally (left). Right image shows a time-lapse movie of the TTG balancer line. Time-lapse movies start during cellularization at stage 5 (i.e. 40 min before furrow closure).

Development • Supplementary information

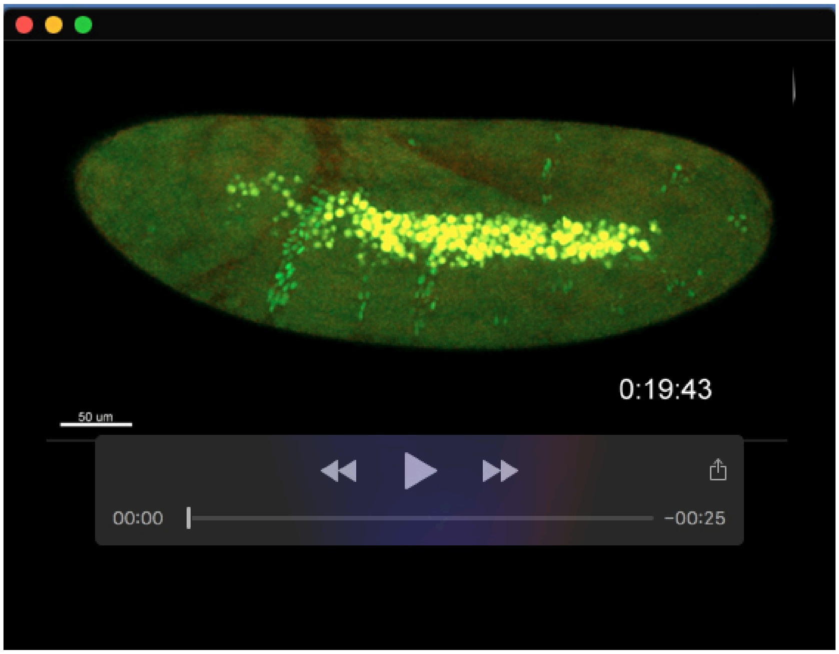

Movie 4. Cell nuclei labelling using NNT construct and En-Gal4 driver. En- Gal4 is driving the expression of the NNT construct with EGFP deposited maternally. Movie starts at stage 8/9.

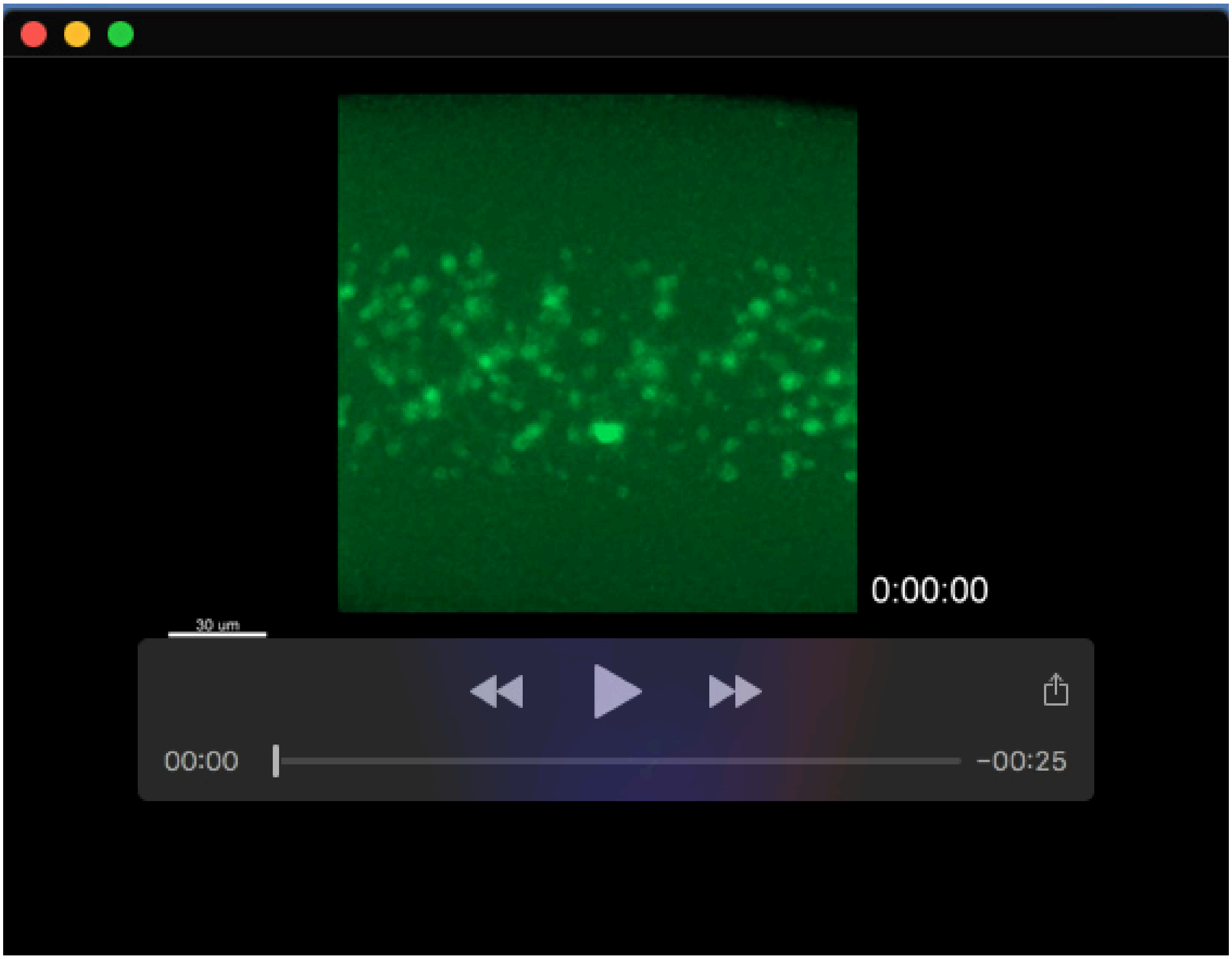

Movie 5. Early cell nuclei labelling (using maternal expressed GFP) combined with late cell nuclei labelling (using zygotically expressed NLS-GFP). 2xPE-Gal4 is driving the expression of the NNT construct (with EGFP deposited maternally) together with NLS-GFP construct to label the mesoderm (using the 5xUAS::NNT, UAS::NLS-GFP fly line). Time-lapse movie starts during cellularization at stage 5 (i.e. 40 min before furrow closure).

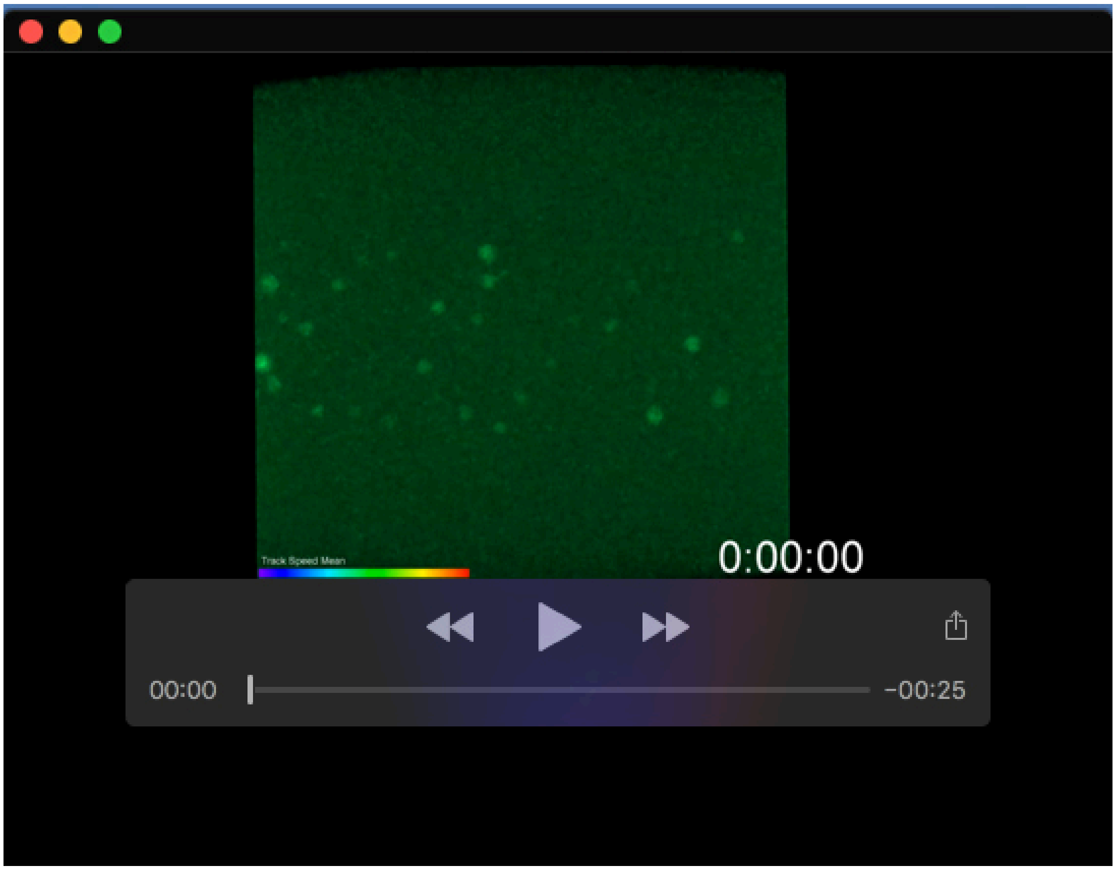

Movie 6. Automatic tracking of cells in the early *Drosophila* embryo in which nuclei are labelled with NaNuTrap method. Cells in Movie 2 (i.e., Kr-Gal4 driving expression of NNT in presence of EGFP deposited maternally) tracked automatically without manual correction using the Imaris software tracking function. Spot detection and tracking can be initiated earlier compared to conventional cell labelling methods as cell nuclei are labelled more effectively using NNT (e.g. see Movie 2). Color bar indicates average speed of the tracks (0-4.5 μm/min).

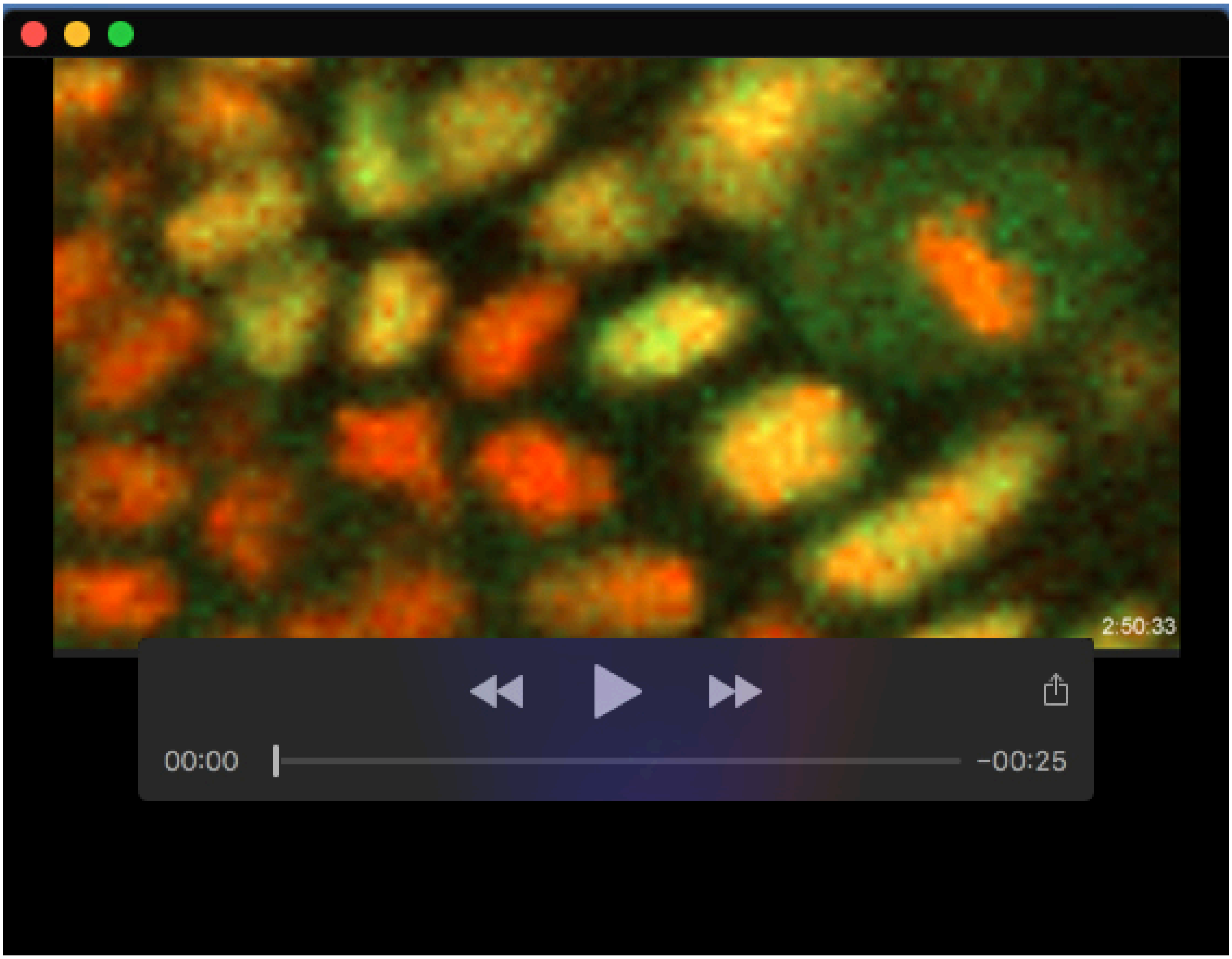

Movie 7. Loss of NNT driven signal is an indicator of cell division. Movie shows dividing mesoderm cells labelled with NNT (green) while histone is labelled in all cells (red, His2Av-mRFP1). To label the mesoderm, we collected embryos from the cross of His2Av-mRFP1; NNT, Vasa::EGFP x Twi-Gal4.
